# Evolution of Milk Consumption and Its Psychological Determinants: A Mini-Review

**DOI:** 10.3389/fnut.2022.845154

**Published:** 2022-02-11

**Authors:** Greta Castellini, Guendalina Graffigna

**Affiliations:** ^1^EngageMinds HUB—Consumer, Food & Health Engagement Research Center, Milan, Italy; ^2^Faculty of Agricultural, Food and Environmental Sciences, Università Cattolica del Sacro Cuore, Cremona, Italy

**Keywords:** milk, consumer psychology, lactose-free, food, determinant

## Abstract

The consumption of lactose-free products and in particular lactose-free milk is increasing worldwide. Although many studies claim that this dietary trend is mainly determined by the number of lactose intolerant people that is growing, others state that most of them self-report an intolerance that has not been diagnosed by medical tests. However, many researchers reported that the consumption of lactose-free milk may put the consumers' health at risk especially when the subjects are not intolerant. Consequently, understanding this new dietary trend considering its main determinants it is necessary to generate educational and intervention campaigns useful to guide people toward healthier and more adequate eating styles. For these reasons we conducted a narrative mini review to summarize the factors contributing to the consumption of lactose-free milk as an alternative to cow's milk, exploring intrinsic and extrinsic product characteristics, biological and physiological, as well as psychological, situational and socio-cultural factors. This narrative mini-review shows that there are six categories of factors that affect the consumption of lactose-free milk. In particular, the intrinsic aspects linked to the product and the socio-demographic characteristics of the consumer are the most explored. On the contrary, situational and socio-cultural factors are the least studied. Finally, this study argues that there are too few studies that investigates the emotional, identity and social aspects underlying these food choices, suggesting the development of future research that investigate the implicit consumer subjective levers to decipher lactose-free milk consumptions.

## Introduction

Milk consumption is definitely declined in the last decades, particularly in developed countries (1). In Italy the consumption of dairy products and milk has been decreasing in a progressive way, from 56.4 L pro capita in 2009 to 50.2 L in 2014 (1). On the other hand, lactose-free dairy market is expected to reach a turnover of 9 billion by 2022 and continues to surpass overall dairy products (7.3 vs. 2.3%) (2). Milk is the dairy category with the highest proportion of lactose-free products, represents two-thirds of the market and determines the growth of the category (2). However, it was demonstrated that this widespread consumption of “lactose-free” products can generate many health problems. In particular, the problematic aspects generated by this eating style are both nutritionally (3) and in terms of quality of life (4), especially when the subjects are not intolerant consumers (5, 6). Given these premises many studies have been carried out to explore this food trend (7, 8) and to understand consumers' attitudes and purchase decisions. Indeed, the identification of consumption determinants is paramount to guide educational and communication interventions to support suitable and health diets. Personal differences, such as knowledge, attitudes habits and socio-demographic factors, are linked to consumers' purchase behaviors increasing the intricacy of decision-making process. These variables are well described and considered by the recent perspective of Köster and Mojet (9). They suggested an interdisciplinary framework to organize and describe the connection among the variables implied in consumer food choice and eating behaviors. This model is particularly innovative and complete in considering the complexity of consumer food choice, and particularly for the case of “free-from” food products (10). Considering the consumption of lactose-free milk, some studies underlined that these changes in milk consumption seem to be more related to the consolidation of new lifestyles and psychosocial variables than simple health reasons such as medical requirement for facing intolerances (11–14). However, there are no studies that try to summarize the main psycho-social factors that lead consumers to prefer consuming lactose-free milk instead of cow's milk. In this narrative mini-review we will contribute to the scientific debate by summarizing the main psycho-social factors contributing to the consumption of lactose free milk as an alternative to cow's milk.

## Materials and Methods

### Search Strategy and Data Analysis

A search for literature investigating the factors that influence the consumption of lactose-free milk was carried out mainly on Scopus and PsycINFO using the keywords “milk”, “lactose-free”, “determinants” and “consumption”. In this narrative mini-review peer-reviewed papers written in English and in Italian related to factors influencing lactose-free milk consumption were considered. A qualitative synthesis of the main determinants of lactose-free milk consumption was conducted, organizing them according to the framework proposed by Köster and Mojet (9). In more details, the model groups the factors that can affect a consumption choice in 6 areas: *psychological factors*, such as cognitive processes, decision making, and personality traits; *situational factors*, such as habits and social signification processes of the context; *sociocultural factors*, like culture, beliefs, and socio-demographic features; *extrinsic product characteristics*, such as brand, labels, and packaging; *intrinsic product characteristics*, such as texture, smell, taste, and nutritional composition; and finally, *biological factors*, such as variables related to consumers' intolerances and immune system functioning.

## Results

### The Main Determinants of the Consumption of Lactose-Free Milk

**Figure 1:**
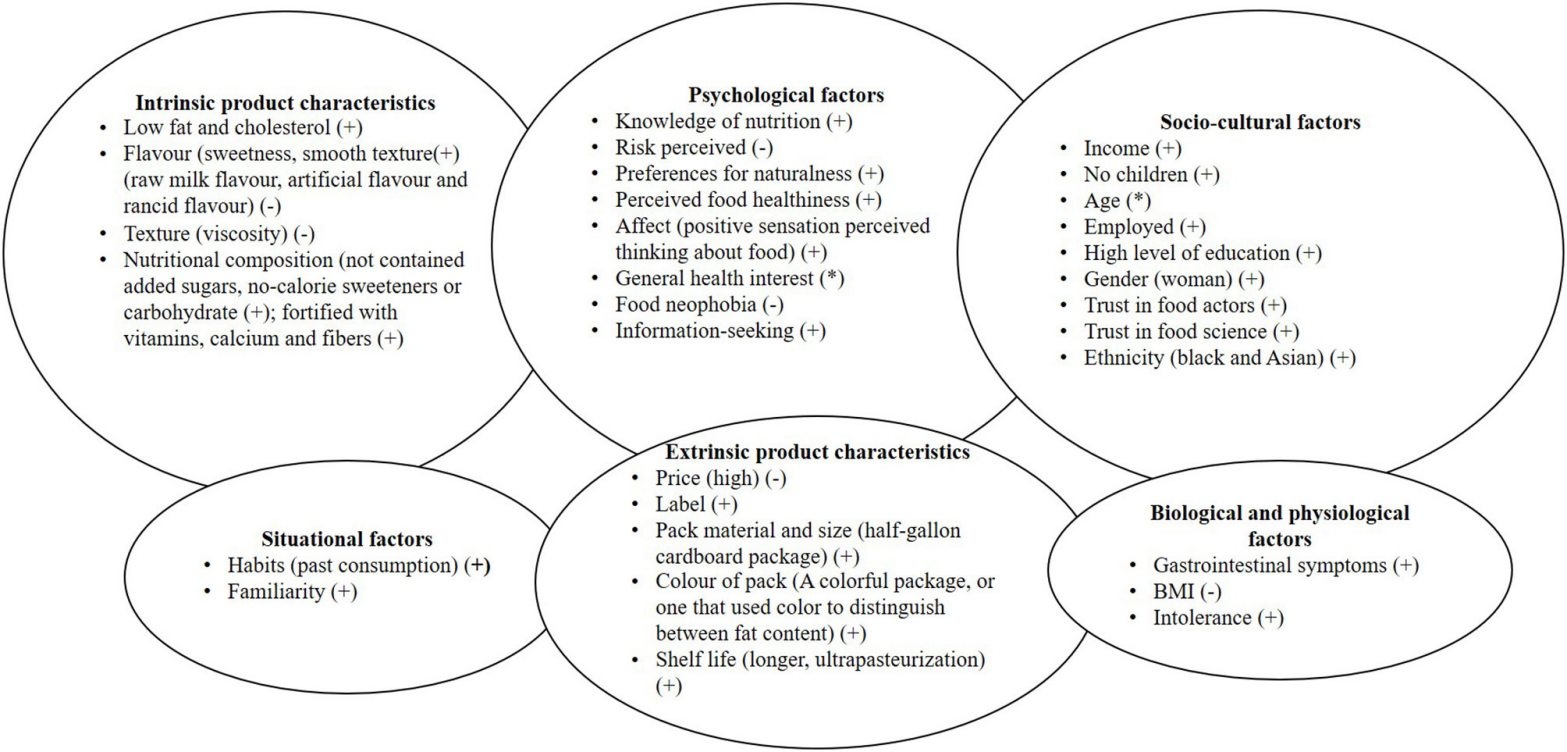
An integrated model of consumer behavior determinants in lactose-free milk consumption. (+), facilitators; (−), barriers; (*), No univocal role.

There are several variables that have been studied to understand the determinants that influence the consumption of lactose-free products and in particular the consumption of milk. As anticipated, we organized them according to the model of Koster and Mojet (9) considering 6 main areas (Figure 1).

#### The Psychological Factors That Influence the Lactose-Free Milk Consumption

Some researchers have focused on understanding the connection between characteristics linked to cognitive processes, decision making and some personality traits and the consumption of lactose-free milk. It has been shown that *nutrition knowledge* and in particular the knowledge of the different nutritional properties of milk can influence the consumption of lactose-free milk (15, 16) especially if consumers *frequently look for information* regarding food nutritional features on food packages (14). Moreover, another important variable in affecting this type of consumption is the *perception of risk* linked to the product and, in particular, the risk of getting cancer. A study carried out on an Italian sample of consumer (1) noted that the non-consumption of milk (with or without lactose) is mainly determined by the expectation to prevent cancer. In addition, *the positive sensation perceived thinking about lactose-free milk (affect)* increases the intention to buy and pay more for it. Moreover, the *perception of product healthiness* and the *preference for natural products* lead to a greater consumption and an enhanced willingness to pay a premium price for lactose-free milk, in different countries such as UK, Sweden, Poland and France (14). Finally, another important variable that in the last years has been often used to explore consumers' intentions or behaviors is *Food Neophobia* (17, 18). Correlating this variable with the consumption of lactose free milk, it can be noticed that those who have a low level of food neophobia are more willing to buy lactose-free milk (19). Finally, a last variable that was studied to map lactose-free milk consumption is the *general health interest* (20). This variable led to conflicting results because some studies show that the general interest in own health increases the consumption of lactose-free milk (19), whereas others found no significant relationship between them (14).

#### The Biological Factors That Influence the Lactose-Free Milk Consumption

Past studies noticed that those who consume a high quantity of lactose-free milk have a *medium-low Body Mass Index* (1) and they often have *gastro-intestinal problems* (21) not always certified as intolerances. However, those who have a *certified intolerance* consume lactose-free milk more frequently (22).

#### The Socio-Cultural and Situational Factors That Influence the Lactose-Free Milk Consumption

If we consider aspects linked to people's socio-demographic characteristics, we notice that *employed women* with a *high educational* qualification and a *high income* consume more lactose-free milk than other targets (1, 13, 15, 16, 21, 22). Moreover, people *without children* and therefore with a family composed by few members are those who consume more frequently lactose-free milk. This study also noticed that *ethnicity* is an aspect that affects the consumption of this product (13). In particular blacks or Asians are those who consume more lactose-free milk. Moreover, there are contrasting results about the role of *age*. Indeed, some researches show that elderly people consume higher quantities of lactose-free milk (16) whereas in other researches elderly people do not consume milk even if without lactose (1) and young people under the age of 30 consume more lactose-free milk (22). The *trust in food actors and in food science* are other variables took into account by past research (14). Some studies show that people with a high level of trust in food science and in food actors are more prone to consume lactose-free milk. Considering situational factors, it is possible to claim that *consumption habits* and *familiarity* with the product affect the consumer behavior, indeed those who are used to consume lactose-free products or plain processed milk are more willing to consume lactose-free milk (19, 20).

#### The Extrinsic Product Characteristics That Influence the Lactose-Free Milk Consumption

The main extrinsic characteristics of lactose-free milk that can affect its consumption are *the price* and *the labels*. In particular, although people are willing to pay more for this product, the price has a negative correlation with the consumption of lactose-free milk (16, 20, 23). In addition, the presence of a known label that guarantees the absence of lactose in the milk increases the intention to purchase it (14, 22). Finally, also the *color, size of the pack and the shelf life* have an impact on lactose-free milk consumption. Ultra-pasteurized lactose-free milk in a half-gallon cardboard package was the ideal (20).

#### Intrinsic Product Characteristics That Influence the Lactose-Free Milk Consumption

Many studies have focused on understanding how the intrinsic characteristics of the product can impact on its consumption. In particular, it has been observed that the consumption of lactose-free milk is mainly determined by the belief that it contains *less fat and cholesterol* than milk with lactose (15). Considering the nutritional composition, consumers prefer lactose-free milk that does not contain *added sugars, calorie sweeteners or carbohydrate* and that is *enriched with vitamins, calcium and fibers* (22, 24). Furthermore, the *flavor* and in particular the sweetness and smooth consistency determine higher level of lactose-free milk consumption (1, 19, 20). On the contrary, grassy *odor*, raw milk flavor, artificial flavor and rancid flavor negatively affect the consumption of lactose-free milk. Moreover, the *texture* and in particular the viscosity of milk can affect its consumption (20). In particular lactose-free milk was preferred to milk with lactose because its viscosity is lower.

## Discussion

The studies carried out on lactose-free milk consumers have investigated different drivers that influence this type of consumption. However, the aspects related to the intrinsic characteristics of products and socio-demographic features of consumers are the most analyzed while psychological and contextual factors are less explored. In particular, this study shows that lactose-free milk consumers have a high socio-demographic profile given by a high income and educational qualification (13, 15, 22). This profiling is in line with other studies carried out on “free from” consumers (25). Another important factor concerns the socio-cultural and situational characteristics of consumers. Those who are more prone to purchase lactose-free milk are people who have a strong trust toward food actors and food science and have a strong familiarity toward this product since they consumed it in the past (14, 20). In addition, these consumers prefer labels on milk which clearly certify the absence of lactose (14, 22). Moreover, if we consider the psychological variables, the study shows that consumers of lactose-free milk declare to be aware of their consumption choices as they have knowledge about food that allow them to choose quality products (15, 16). They are also particularly attentive about food choices indeed they frequently consult labels in order to understand the nutritional qualities of products (14, 24). However, we note that there are emotional components that influence the consumption of lactose-free milk both as barriers and as facilitators of consumption (1, 14). In particular, research conducted by Hartmann et al. (14) showed that the positive emotional reaction (affect influence), triggered by the label “lactose-free”, is an aspect that positively influences the consumption of lactose-free milk. The consumer, in fact, believes that this “free-from” label is synonymous with healthiness, encouraging the purchase of lactose-free milk. Many studies carried out on “free-form” labels (26, 27), have noted that these open up a positive psychological dimension in consumers even in the absence of risk information on the eliminated ingredient. These studies highlight an unconscious psychological mechanism that the “free-from” label generates in consumers: they perceive the removed ingredient as risky just because it has been removed (according to the simplifying mental equation that “if it is removed it is because is dangerous”) and the positive emotion given by the avoidance of a possible health risk leads them to a greater willingness to purchase “free-from” products and in particular lactose-free milk. These findings underline that the perception of risk is an important emotional barrier that influence the consumption of milk in general. Indeed, some Italian consumers (1), even though they believe that lactose-free milk is healthier than milk with lactose, it is equally perceived as harmful to health and therefore avoided.

All these findings seem to describe an aware and informed consumer, attentive to his/her food choices with a high socio-demographic profile that gives him/her the economic and cultural power to make accurate and conscious consumer choices. However, by applying a psychological lens to the reading of the data, we can argue that this consumer is strongly affected by emotions that drive the decision-making process. Consumers, in fact, tend to demonize lactose considering it as an ingredient to be avoided and emotionally linked to negative feelings and health risk. Moreover, the awareness and food knowledge declared by lactose-free milk consumers is mainly aspirational since it was scientifically demonstrated that the consumption of this product can determine health risks, especially when the subjects are not intolerant consumers (5, 6). These results show how consumers of lactose-free milk aspire to be aware of their choices and that they have a need to control them, wanting to play a leading role in their food consumption by choosing products that support this identity image such as lactose-free milk. Indeed, as already showed by past studies (7) the purchase of “free from” products is a means that allow consumer to express their affirmation and their control on food choices, showing their active and critical role as consumer. “Free-from” food choices, therefore, are strongly governed by emotional, identity and psychological aspects linked to a need for self-affirmation and self-expression rather than rational and conscious processes. As pointed out by other research, food choices and particular dietary styles are means used by people to establish social connections (28), to express own identity in order to be accepted by others (29, 30). Hence, the subjective meaning given to food plays a key role in the purchase of it. Furthermore, as demonstrated by other researchers, social influence considered as imitation, group belonging, and social identification, can play an important role in determining food choices and especially the “free-from” ones (31). Indeed, as showed by Xhakollari et al. (31), the fact that family members or friends believe that it is right to follow a gluten-free diet increases the likelihood that the person follows this eating style, especially if the choice is voluntary and not determined by certified intolerances. However, there are too few research on the consumption of lactose-free milk that investigates the emotional, identity and social factors underlying these food choices. This highlights the lack of consumer psychology studies on the topic of “free-from” consumption that should be deepen. This unexplored field of research should be covered by carrying out research that investigates the relationship between lactose-free milk consumption and the individual and social dimensions of consumers because only in this way it is possible to understand what are the most important determinants of this consumption, creating communication processes that guide people toward healthier diets.

## Author Contributions

GC: conceptualization, methodology, and writing—original draft. GG: writing—review and editing and supervision. Both authors have approved the final article.

## Funding

This work was supported by the Fondazione Cariplo and Regione Lombardia within the CRAFT (Cremona Agri-Food Technologies) project ID 2018/2757.

## Conflict of Interest

The authors declare that the research was conducted in the absence of any commercial or financial relationships that could be construed as a potential conflict of interest.

## Publisher's Note

All claims expressed in this article are solely those of the authors and do not necessarily represent those of their affiliated organizations, or those of the publisher, the editors and the reviewers. Any product that may be evaluated in this article, or claim that may be made by its manufacturer, is not guaranteed or endorsed by the publisher.
